# Associations of Undergoing a Routine Medical Examination or Not with Prevalence Rates of Hypertension and Diabetes Mellitus: A Cross-Sectional Study

**DOI:** 10.3390/ijerph13070628

**Published:** 2016-06-23

**Authors:** Lin Zhang, Wei Zhang, Lingling Zhang, Danping Tian, Li Li, Xin Deng, Jing Deng, Peishan Ning, Guoqing Hu

**Affiliations:** 1 Department of Epidemiology and Health Statistics, Xiangya School of Public Health, Central South University, 110 Xiangya Road, Changsha 410078, China; zhanglinxysm@163.com (L.Z.); zwzhangweizw@gmail.com (W.Z.); danpingtian@foxmail.com (D.T.); cslilee1009@gmail.com (L.L.); dengxin66@126.com (X.D.); dengjing2@126.com (J.D.); ningpeishan033@163.com (P.N.); 2 Department of Public Health Sciences, Clemson University, Clemson, SC 29634, USA; lingliz@clemson.edu

**Keywords:** prevalence, hypertension, diabetes mellitus, household interview survey, China

## Abstract

Background: Undergoing a routine medical examination may be associated with the prevalence rate of chronic diseases from a population-based household interview survey. However, this important issue has not been examined so far. Methods: Data came from the first health service household interview of Hunan province, China, in 2013. A Rao–Scott chi-square test was performed to examine the difference in prevalence rates between subgroups. Adjusted odds ratio (OR) was calculated using the PROC SURVEYLOGISTIC procedure of SAS9.1 statistical software. Results: In total, 24,282 residents of 8400 households were surveyed. A higher proportion of elderly adults had undergone a medical examination within the prior 12 months compared with young adults (≥65 years, 60%; 45–64 years, 46%; 18–44 years, 37%). After controlling for location, sex, and household income per capita, undergoing a medical examination was significantly associated with high prevalence rates of hypertension (adjusted OR: 2.0, 95% CI: 1.1–3.5) and of diabetes mellitus (adjusted OR: 3.3, 95% CI: 1.7–6.5) for young adults aged 18–44 years. The associations were not statistically significant for age groups 45–64 years and 65 years or older. Conclusion: The prevalence rates of hypertension and diabetes mellitus may be seriously underestimated for young adults not undergoing a routine medical examination in a health household interview survey.

## 1. Introduction

It has been estimated that chronic diseases caused a loss of 1.34 billion disability-adjusted life years (DALYs) in 2010 [1], and nearly half of the loss was caused by years lived with disability (YLDs) [2]. Hypertension and diabetes mellitus are two common causes of chronic diseases. Reliable morbidity rates (typically prevalence) are important inputs of the calculation of local, national, regional, and global DALYs. However, many countries do not have high-quality prevalence data of chronic diseases [3].

A household interview survey is a common method of collecting prevalence data, in addition to population- or hospital-based disease registries [3,4]. Many countries adopt household interview surveys to estimate the prevalence rates of chronic diseases [5]. In household interview surveys, the identification of cases of chronic diseases is often based on a diagnosis by a physician in the prior 6 months or longer [6,7,8].

Chronic diseases develop slowly and usually are asymptomatic or have few symptoms in early stages (typically in young adults) [9]. Young adults with no symptoms or few symptoms may not realize that they have a chronic disease if they do not undergo a medical examination [10]. Previous studies show low proportions of residents undergoing medical examination in some countries—18.8% in Chinese aged ≥35 years in 2008 [8], 20.9% in Americans aged ≥18 years in 2002–2004 [11], and 46.8% in Taiwanese aged ≥65 years in 2005 [12]. This may lead to the underestimation of the prevalence of chronic diseases, further affecting the assessment of chronic disease burden. Unfortunately, this issue has not been examined so far.

The first health service household interview survey of Hunan province, China, was completed in 2013, which included information of chronic diseases diagnosed in the prior 6 months and that of “undergoing a medical examination in the last year or not”. Therefore, this survey allowed us to examine the association of undergoing a medical examination or not with the prevalence rates of hypertension and diabetes mellitus.

### Objectives

Using data of the first health service household interview survey of Hunan province, China, we examined the primary research assumption—that the prevalence rates of hypertension and diabetes mellitus are higher in young adults undergoing a medical examination than in young adults not undergoing a medical examination.

## 2. Methods

### 2.1. Data Source

Data of this study were obtained from the first health service household interview survey of Hunan province, which was organized by the Center for Health Education and Information Statistics, Provincial Health and Family Planning Commission of Hunan (former Hunan Health Bureau). A multi-stage random sampling was used in this survey to obtain 24 282 residents of 8400 households (4200 from urban areas and 4200 from rural areas). First, we selected 14 counties from 14 municipalities (one county per municipality); seven countries were used to obtain an urban sample (targeting urban residents) and the other seven countries to obtain a rural sample (targeting rural residents). Second, we randomly chose 5 towns in each selected county. Third, 2 villages or communities were selected at each given town at random. Last, 60 households were selected at random in each village or community, and the family members of these 60 households formed the study subjects for face-to-face interviews. Data were collected through face-to-face household interviews, performed by trained persons using the standardized questionnaire of the fifth National Health Service Household Interview Survey [13]. A group of experts were invited to inspect the implementation of household interviews at all 14 sample counties (1–2 days per county), identifying the problems in face-to-face interview and providing solutions to them [14]. The survey proposal was approved by the Provincial Health and Family Planning Commission of Hunan.

During the survey, the interviewers explained the purposes and confidentiality of the survey, and then invited family members to participate; residents could decline the invitation [13]. Questions of chronic diseases were only proposed to residents aged ≥18 years.

Survey data were de-identified, entered, and checked using the standardized dataset and procedure [13]. A study analysis was approved by the medical ethnic committee of Central South University (XYGW-2016-12).

### 2.2. Outcome Measures

Residents with chronic diseases were defined as “the persons (who) had any chronic disease diagnosed by physicians in the prior 6 months, or had diagnosed chronic diseases over 6 months (previously) but were receiving medications or physical therapy when interviewed”. The interviewers first asked the interviewee whether he or she had hypertension and diabetes mellitus diagnosed by physicians in the prior 6 months. Then, the interviewers asked the interviewee whether he or she had other chronic diseases diagnosed by physicians in the prior 6 months using the classification of diseases recommended by the fifth National Health Service Household Interview Survey [13]. A total of 75 chronic diseases were included in the survey questionnaire. We focused on hypertension and diabetes mellitus, defined as the 10th International Classification of Diseases (ICD-10) code of I10–I15 and E10–E14, respectively [15].

### 2.3. Independent Variables

The question, “Did you undergo a medical examination in the prior 12 months (excluding the examinations for purposive medical diagnosis and treatments)?” was used to measure whether a resident underwent a medical examination or not.

We selected location (urban/rural), sex, age group, and household income per capita as covariates given that they were reported being associated with the prevalence of hypertension and diabetes mellitus [16,17,18]. Age was grouped into three groups: 18–44 years, 45–64 years, and ≥65 years due to the similarity of prevalence between age groups (data not shown here).

We equally divided the households into five categories based on the household income per capita in the last year for urban areas and rural areas separately: lowest (urban, <6667 Yuan; rural, <3334 Yuan); lower (urban, 6667–9999 Yuan; rural, 3334–4999 Yuan); average (urban, 10,000–14,999 Yuan; rural, 5000–7499 Yuan); higher (urban, 15,000–23,999 Yuan; rural, 7500–9999 Yuan); and highest (urban, ≥24,000 Yuan; rural, ≥10,000 Yuan).

### 2.4. Statistical Analysis

The prevalence rate was calculated as “number of persons having diagnosed diseases divided by total number of residents × 100%”.

Relative difference in prevalence rates was used to quantify the impact of undergoing a medical examination on the prevalence of chronic diseases, which was calculated as “(the prevalence of residents undergoing a medical examination—the prevalence of residents not undergoing a medical examination)/the prevalence of residents undergoing a medical examination × 100%”.

A Rao–Scott chi-square test was performed to examine the statistical significance of the relative difference in prevalence rates. To control the effects of covariates (location, sex, and household income per capita), we ran multivariate logistic regression by age group to estimate the impact of not undergoing a medical examination on the prevalence rates of hypertension and diabetes mellitus. *p* < 0.05 was considered to be statistically significant. Sampling weights were taken into account for data analysis by using the PROC SURVEYFREQ procedure and the PROC SURVEYLOGISTIC procedure of SAS9.1 statistical software [19].

## 3. Results

### 3.1. Proportion of Residents Undergoing a Medical Examination

Of 24 282 participants, 19 320 (79.6%) were 18 years or older (Table 1). Urban residents constituted 51.0% of participants. 45.6% of adults aged ≥18 years reported undergoing a medical examination in the prior 12 months in Hunan province. Older adults had a higher likelihood of undergoing a medical examination than young adults in the prior 12 months (45–64 years vs. 18–44 years, OR: 1.4; ≥65 years vs. 18–44 years, OR: 2.5). The proportion of undergoing a medical examination was a little higher in females than in males (47.4% vs. 43.7%).

### 3.2. Prevalence Rates of Hypertension and Diabetes Mellitus

The prevalence rates of hypertension and diabetes mellitus were higher in persons undergoing a medical examination than in persons not undergoing a medical examination for all adults aged ≥18 years (hypertension: 15.5% vs. 9.6%; diabetes mellitus: 3.6% vs. 2.1%) (Table 2). Similar differences were observed in all location-, sex-, age- and income-specific groups, except for diabetes mellitus prevalence in the highest household income group, although the gaps in some groups were insignificant.

### 3.3. Associations of Undergoing a Medical Examination with Chronic Prevalence Rates

After controlling location, sex, and household income per capita, undergoing a medical examination was significantly associated with high hypertension prevalence (adjusted odds ratio: 2.0, 95% CI: 1.1–3.5) and high diabetes mellitus prevalence (adjusted odds ratio: 3.3, 95% CI: 1.7–6.5) for age group 18–44 years (Table 3). The associations of undergoing a medical examination with the prevalence rates of hypertension and diabetes mellitus were not statistically significant for age groups 45–64 years and ≥65 years.

## 4. Discussion

### 4.1. Main Findings of This Study

Using the provincial health household interview survey data of Hunan, we report here for the first time that high prevalence rates of hypertension and diabetes mellitus in adults aged 18–44 years were significantly associated with undergoing a medical examination in the prior 12 months.

The results also reflect that over 50% of residents younger than 65 years did not undergo a medical examination in the last year regardless of location of residents, sex, and household income.

### 4.2. What is Already Known for This Topic

Compared with low proportion of undergoing a medical examination among Chinese aged 35 years and older in 2008 (18.8%) [8], the proportion increased substantially for young adults. The large difference may be mainly due to the implementation of the National Basic Public Health Service Package and other public health efforts. Since 2009, China has initiated a national health promotion program, which provides free public health services to all residents [20]. The higher proportion of elderly adults aged ≥65 years undergoing medical examinations is probably due to free government-provided medical examination for the elderly [21] and mild or severe symptoms of chronic diseases among the elderly [22]. Poor health awareness partially explains why fewer young persons undergo a medical examination. A study by Xue et al. reported that the health awareness of the residents was very low at Taian, China, although the prevalence of chronic kidney disease was high [23].

The differences in prevalence gaps between persons undergoing a medical examination and those not undergoing a medical examination from location, sex, and household income per capita may reflect differences in the content and quality of medical examination. For example, the medical examination conducted in urban areas is usually more comprehensive, and the service quality is better than that conducted in rural areas due to the uneven allocation of health resources [24,25,26]. Compared with rural residents, many employed urban residents regularly undergo an extensive health examination [27], increasing the likelihood of having certain diseases such as hypertension and diabetes mellitus detected.

### 4.3. What This Study Adds

The results confirm our assumptions, strongly suggesting that the prevalence rates of hypertension and diabetes mellitus may be seriously underestimated for young adults who do not undergo a medical examination. These findings have not been previously reported. As we mentioned above, the results are primarily due to two factors: (a) young adults are at early stage of hypertension and diabetes mellitus, having no symptoms or few symptoms; and (b) young adult patients with no symptoms or few symptoms rarely undergo medical examination for chronic diseases. This indicates that patients who have no symptoms or few symptoms of hypertension and diabetes mellitus are less likely to be detected and treated early if they do not undergo a routine medical examination.

There are two important implications of this study. First, the impact of not undergoing a medical examination should be taken into consideration when using the household interview data to estimate the prevalence rates of hypertension and diabetes mellitus for young adults in countries where many residents do not undergo a regular medical examination. This also applies to other chronic diseases, such as chronic obstructive pulmonary disease with no symptoms or few symptoms in early stages. Second, regular medical examination should be promoted or even mandated for young adults in order to detect, diagnose, and treat chronic diseases that have no symptoms or few symptoms early. It has been well-known that early detection and early treatment of chronic diseases are critical for reducing global chronic disease burden and narrowing health disparities from chronic diseases between developed countries and developing countries [3].

### 4.4. Limitations of This Study

Our findings are mainly limited by the absence of details of medical examination that residents underwent in the prior 12 months. Without the details of medical examination in the prior 12 months, we cannot assess whether the diagnostic measures of hypertension and diabetes mellitus (e.g., blood pressure and blood glucose) were included in the medical examination, whether the diagnoses were based on clinical criteria (systolic and diastolic blood pressure, blood glucose), or how many cases were identified through a routine medical examination. Moreover, we cannot determine whether the medical examination occurred earlier or later than the detection of chronic diseases since the time periods of undergoing a medical examination and being diagnosed with a chronic disease were inconsistent, which may affect our results to some extent. This issue needs to be assessed in future studies, which can be used to adjust the prevalence rates obtained from a household interview survey. Other factors such as family history of disease may also influence the results because people with a family history of disease may have a higher chance of undergoing a medical examination than those without a family history of disease. Due to a lack of high-quality and standardized exposure data on occupation, smoking, and alcohol consumption, we did not include these three variables as confounding factors to control for their impacts on our results. In addition, we did not analyze the impact of not undergoing a medical examination on the prevalence rates of other chronic diseases because of the insufficient sample size. Similar analysis for other chronic diseases could be performed using the national health household interview data.

## 5. Conclusions

We conclude that the prevalence rates of hypertension and diabetes are very likely underestimated for young adults who do not undergo a medical examination regularly. This issue should be taken into account when estimating the burden of diseases at local, national, regional, and global levels. Further studies are needed to quantify the extent of underestimation, thus providing the base of necessary adjustment to household interview data. In addition, the routine medical examination of chronic diseases should also be promoted among young adults to promote early detection and early treatment of chronic diseases.

## Figures and Tables

**Table 1:** Proportion of residents undergoing a medical examination (ME) in the prior 12 months (Hunan of China, 2013).

Variable	*N*	Residents Undergoing ME	Proportion (95% CI)	Odds Ratio (95% CI)
Total	19,320	8703	45.6 (27.6–63.6)	NA
Location				
Urban	9894	4325	47.2 (38.5–55.9)	1.0
Rural	9426	4378	45.1 (21.8–68.4)	0.9 (0.4–2.3)
Sex				
Male	9472	4089	43.7 (24.9–62.5)	1.0
Female	9848	4614	47.4 (30.1–64.8)	1.2 (1.1–1.3) *
Age group				
18–44 years	6889	2546	37.4 (17.5–57.3)	1.0
45–64 years	8620	3893	46.2 (28.8–63.5)	1.4 (1.2–1.8) **
≥65 years	3811	2264	59.6 (43.6–75.5)	2.5 (1.7–3.6) **
Household income Per Capita ^#^				
Lowest	4894	2493	42.7 (26.3–59.0)	1.0
Lower	3519	1690	44.1 (32.7–55.4)	1.1 (0.9–1.3)
Average	4312	1850	41.3 (23.0–59.6)	0.9 (0.8–1.1)
Higher	2757	1142	47.8 (29.3–66.4)	1.2 (1.0–1.5) *
Highest	3784	1511	49.4 (28.0–70.8)	1.3 (0.9–1.8)

CI: confidence interval. ^#^: The incomes of some households were missing; Household income per capita included five categories: lowest (urban, <6667 Yuan; rural, <3334 Yuan); lower (urban, 6667–9999 Yuan; rural, 3334–4999 Yuan); average (urban, 10,000–14,999 Yuan; rural, 5000–7499 Yuan); higher (urban, 15,000–23,999 Yuan; rural, 7500–9999 Yuan); and highest (urban, ≥24,000 Yuan; rural, ≥10,000 Yuan). *: *p* < 0.05; **: *p* < 0.01. Note: The sum of sample sizes of subgroups was a little smaller than 19,320 because of the presence of missing values. Missing values were excluded from statistical analysis.

**Table 2:** Prevalence rates of hypertension and diabetes mellitus in residents undergoing a medical examination and not (Hunan of China, 2013).

Variables	Hypertension			Diabetes Mellitus		
	A (%)	B (%)	(A − B)/A × 100%	A (%)	B (%)	(A − B)/A × 100%
Total	15.5	9.6	38% *	3.6	2.1	42% *
Location						
Urban	20.7	10.4	50% *	7.2	3.4	53% **
Rural	13.9	9.4	32%	2.5	1.7	32%
Sex						
Male	15.5	9.3	40%	3.5	2.2	37%
Female	15.6	9.9	37% **	3.8	2.0	47% **
Age group						
18–44 years	1.8	1.0	44% **	1.1	0.4	64% **
45–64 years	16.2	12.5	23%	3.9	2.6	33%
≥65 years	30.4	25.7	15%	6.1	5.2	15%
Household Income Per Capita ^#^						
Lowest	16.7	11.8	29%	3.9	1.4	64% **
Lower	15.3	9.3	39% **	3.6	1.1	69% **
Average	15.1	9.0	40%	3.6	1.8	50%
Higher	15.2	9.9	35%	4.9	1.8	63% *
Highest	15.6	8.8	44% *	2.9	3.2	−10%

A: persons undergoing a medical examination in the prior year; B: persons not undergoing a medical examination in the prior year. ^#^: The incomes of some households were missing. Household income per capita included five categories: lowest (urban, <6667 Yuan; rural, <3334 Yuan); lower (urban, 6667–9999 Yuan; rural, 3334–4999 Yuan); average (urban, 10,000–14,999 Yuan; rural, 5000–7499 Yuan); higher (urban, 15,000–23,999 Yuan; rural, 7500–9999 Yuan); and highest (urban, ≥24,000 Yuan; rural, ≥10,000 Yuan). *: *p* < 0.05; **: *p* < 0.01.

**Table 3:** Associations of undergoing a medical examination (ME) with prevalence rates of hypertension and diabetes mellitus (Hunan of China, 2013).

Age Group	Undergoing ME or Not	Hypertension	Diabetes Mellitus
18–44 years	No (reference)	1.0	1.0
	Yes	2.0 (1.1–3.5) *	3.3 (1.7–6.5) **
45–64 years	No (reference)	1.0	1.0
	Yes	1.3 (0.8–2.1)	1.4 (0.9–2.2)
≥65 years	No (reference)	1.0	1.0
	Yes	1.2 (0.9–1.6)	1.1 (0.8–1.5)

The effects of location, sex, and household income per capita were controlled using multivariate logistics regression. *: *p* < 0.05; **: *p* < 0.01.
